# Application of Multi-Sensor Data Fusion and Machine Learning for Early Warning of Cambrian Limestone Water Hazards

**DOI:** 10.3390/s25226854

**Published:** 2025-11-10

**Authors:** Hang Li, Yijia Li, Wantong Lin, Huaixiang Yang, Kefeng Liu

**Affiliations:** 1 Inner Mongolia Research Institute, China University of Mining and Technology (Beijing), Beijing 100083, China; bqt2400101006@student.cumtb.edu.cn (Y.L.); l2206884090@163.com (W.L.); cumtbyanghx@163.com (H.Y.); lkf13233024069719@163.com (K.L.); 2 School of Energy and Mining Engineering, China University of Mining and Technology (Beijing), Beijing 100083, China

**Keywords:** Cambrian limestone aquifer, microseismic-water inrush volume model, genetic algorithm optimization, indicator weight distribution, floor water inrush early warning

## Abstract

The issue of water disasters in the mining floor is extremely severe. Despite significant progress in the on-site monitoring and identification of water inrush channels, research on the spatial development characteristics of cracks and the temporal evolution patterns remains insufficient, resulting in the incomplete development of microseismic-based water disaster early warning theory and practice. Based on this, the present study first derives the expressions for the diameter and length of water inrush channels according to the damage characteristics of microseismic events and the glazed porcelain shape features of the channels. A theoretical model for the correlation between microseismic-water inrush volume is established, and the relationship between microseismic and water level is revealed. Analysis of field monitoring data further indicates that when high-energy microseismic features (such as single high-energy events and higher daily cumulative energy) are detected, the aquifer water level begins to decline, followed by high water inrush events. Therefore, a decrease in water level accompanied by high-energy microseismic features can serve as an important early warning marker for water disasters. Finally, advanced machine learning methods are applied, in which the optimal index combination for floor water inrush prediction is obtained through the genetic algorithm, and the weights of each index are determined by integrating the analytic hierarchy process with the random forest model. Field engineering verification demonstrates that the integrated early warning system performs significantly better than any single monitoring indicator, and all high-water-inrush events are successfully predicted within four days.

## 1. Introduction

Numerous geological safety hazards are encountered during mining, among which floor water disasters are consistently regarded as a critical challenge for the transformation and upgrading of the coal industry [1,2]. Unlike shallow aquifers, the Cambrian limestone aquifer in deeper strata is associated with larger water inrush volumes and higher risks, and it has gradually been identified as a major focus and difficulty in current research [3,4,5]. Therefore, systematic theoretical research and risk level identification of floor water inrush disasters are urgently required, so that the safe, efficient, and sustainable utilization of mineral resources can be effectively ensured [6,7,8].

In terms of the mechanisms of water inrush disasters, extensive research has been carried out on rock mass stability and fracture development [9,10]. This includes studies in which the relationship between microseismic (MS) activity and damage-induced fracturing is investigated using three-dimensional finite element methods, through which real-time monitoring of fracturing events is achieved, providing an important means for assessing the risk of rock mass failure [11,12,13]. Other studies have shown that energy parameters obtained from MS monitoring can effectively characterize the degree of rock mass failure and the state of crack propagation [14]. Therefore, the development of comprehensive sensitivity precursors based on MS energy is considered highly significant for reducing the risk of floor water disasters in coal mines. Meanwhile, some researchers combine MS characteristics with numerical simulation results, and information features and distribution patterns of internal fractures in surrounding rocks are determined through mutual validation [15,16]. In addition, MS parameters combined with intelligent methods are used to estimate the geometry of fractures and their interactions, thereby allowing the distribution of water pressure within fractures and the dominant seepage channels to be obtained [17,18,19]. Other studies indicate that MS mechanisms, when integrated with stress variation, can be employed to identify explicit and implicit geological structures in mines in advance, thus providing safety assurance for underground excavation and mining [20,21]. Moreover, MS monitoring results show that under the reinforcement of single-stage or multi-stage grouting, floor fractures can be effectively filled and the degree of floor damage in coal seams is mitigated [22].

On the other hand, studies on water disaster early warning include the analysis of spatial indicators from MS monitoring data, through which the depth of floor damage during coal seam mining above confined water is determined, allowing a preliminary assessment of water inrush risk in the coal seam floor [23]. A floor water inrush evaluation method is established by calculating weights using mathematical formulas, based on water inrush volume, water content, and pre-mining MS parameters, and the working face is thereby divided into zones with different levels of water inrush risk [24,25]. Based on the dominant frequency components of MS water inrush signals, the variational mode decomposition (VMD) method is applied to effectively extract their high-frequency components, from which the water inrush risk characteristics of the study area are obtained [26,27]. By integrating fracture signals, physical analysis, and simulation modeling, significant variations in the hydraulic characteristics within water inrush channels are revealed, thereby enabling the early warning of coal mine water inrush disasters [28]. In addition, advanced intelligent methods such as SIOA, ResNet, and ISMOTE are introduced to accurately identify fundamental parameters of water inrush sources, and their effectiveness is demonstrated [29,30,31].

Existing studies have achieved certain progress in the risk assessment of floor water disasters [32,33,34], but research on the spatial morphological characteristics of fractures and their temporal evolution prior to water inrush remains insufficient, and the accuracy of related early warning methods still needs improvement. Based on this, the main scientific questions and research objectives of this study include: (1) Developing a theoretical coupling model between MS activity and water inrush volume; (2) Analyzing the co-evolution patterns of MS high-energy features and the decline in Cambrian water levels based on monitoring data; (3) Constructing an intelligent early-warning framework that integrates genetic algorithm (GA)–analytic hierarchy process (AHP)–random forest (RF), achieving precise prediction of floor water hazards through global feature optimization and importance weight distribution.

## 2. Methodology

**Figure 1:**
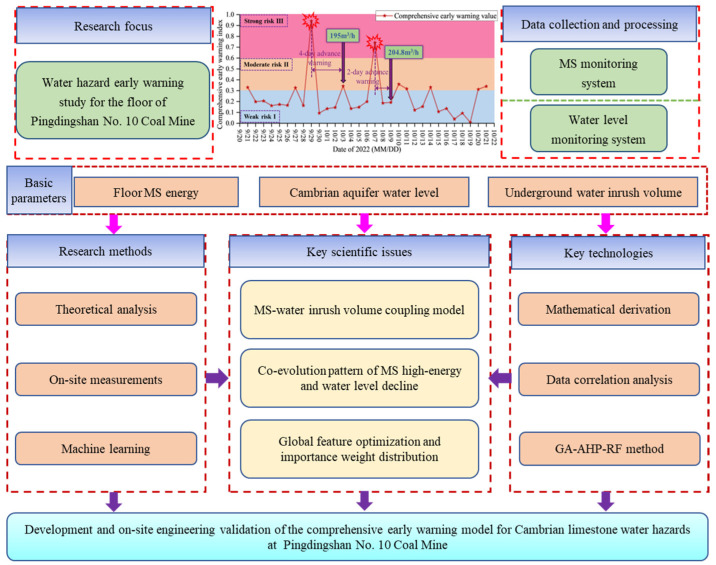
Flowchart of research technology roadmap.

The overall technical roadmap of this study is shown in Figure 1. The process includes steps such as the development of theoretical models, feature correlation analysis, indicator optimization and weight distribution, and comprehensive early warning system validation. The research objective is to develop a comprehensive early-warning system for Cambrian limestone water hazards at Pingdingshan No. 10 Coal Mine, integrating physical information and intelligent algorithms. The specific details are as follows:

(1) Theoretical model development: Based on the MS source damage characteristics, the geometric features of the water inrush channels are derived, and an MS-water inrush volume correlation model is established to provide a theoretical basis for subsequent analyses.
(2) Feature correlation analysis: Representative MS energy indicators (single high-energy events and high daily cumulative energy) and water level change indicators are extracted to identify significant correlation response patterns before water inrush events.
(3) Indicator optimization and weight distribution: GA is used for global optimization of multi-source feature combinations to select the optimal indicator set. The integration of AHP and RF models is used to assign weights to indicators through a combination of subjective and objective methods.
(4) Comprehensive early warning system validation: A water hazard early warning system is constructed based on the GA-AHP-RF fusion algorithm. The model is validated using actual measurement data from the working face of Pingdingshan No. 10 Coal Mine to evaluate its accuracy and timeliness.

## 3. Engineering Conditions and Integrated Monitoring System

### 3.1. Geological Overview

**Figure 2:**
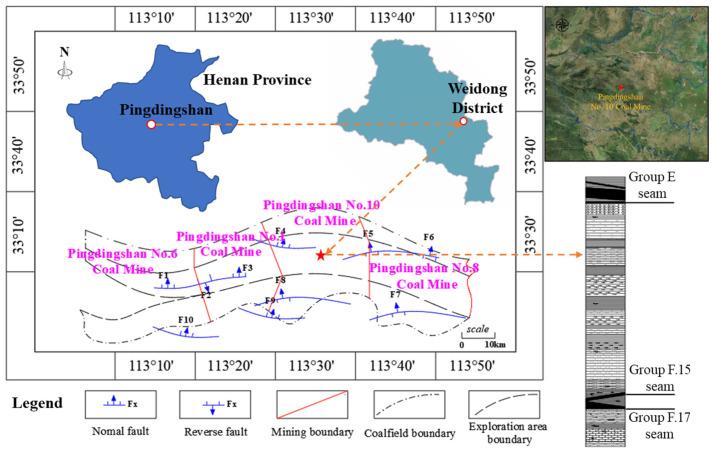
Geographical location map of Pingdingshan No. 10 Coal Mine.

The Pingdingshan No. 10 Coal Mine is located about 5 km from the center of Pingdingshan City, Henan Province, China. The basic structure of its mining area is composed of the No. 10 Coal Mine syncline and the Guozhuang anticline, and its geographical location is shown in Figure 2. The mining area borders the Pingdingshan No. 1 Coal Mine to the east and the Pingdingshan No. 8 Coal Mine to the west. The strike length of the field extends about 4 km from east to west, with a dip width of approximately 5.1 km from north to south. The coal seams of Pingdingshan No. 10 Coal Mine are sequentially named from Group A to Group F from top to bottom. The main coal seams being mined in the area, Group E and Group F, belong to the Permian coal-bearing strata. The floor elevation of Group E is approximately −650 m, while the floor elevation of Group F is approximately −800 m. The F.15 coal seam is located above the F.17 coal seam and was fully mined in 2021. During the extraction of the overlying seam, floor failure fractures and water inrush channels had already formed, resulting in a large number of inrush points at that time. Therefore, field investigations were carried out before the mining of the F.17 seam. The resistance to water pressure is reduced in areas with special geological structures or weak aquicludes, and the possibility of water inrush during mining still exists.

### 3.2. Working Face Overview and Monitoring System Layout

The ground elevation ranges from 140 m to 240 m, while the coal seam elevation ranges from −888 m to −963 m, indicating that the mine is operated at a depth of approximately one kilometer. The thickness of the F.17-33200 working face ranges from 1.2 m to 2.9 m, with an average thickness of 2.4 m. The working face is mined under pressure-bearing conditions, and the Cambrian limestone is the primary confined aquifer in Pingdingshan No. 10 Coal Mine, characterized by significant burial depth, thickness, and well-developed dissolution structures. The lithology is predominantly thick-bedded limestone, with localized interbeds of dolomitic limestone and clayey limestone. Affected by long-term tectonic movements and groundwater dissolution, boreholes have revealed caverns as high as 8.0 m, forming a typical fracture-karst composite aquifer system. The formation of such karst geomorphology significantly enhances water inrush connectivity. Once the floor’s water-proofing layer is damaged by mining disturbances, the pre-existing karst channels can rapidly become the main water inrush pathways, leading to a sharp increase in water inrush from the floor.

**Figure 3:**
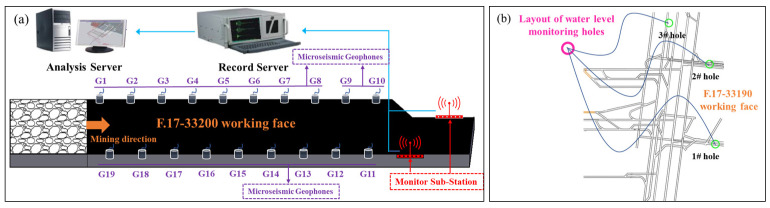
Layout of the monitoring system. (**a**) MS monitoring system; (**b**) Water level monitoring system.

At Pingdingshan No. 10 Coal Mine, water level monitoring boreholes (Borehole 1 and Borehole 2) are installed in each of the ventilation roadway floor and the conveyor roadway floor of the F.17-33190 working face. The Borehole 3 is installed in the central main roadway, enabling real-time dynamic observation of water level changes in the floor aquifer. Simultaneously, an MS monitoring system is introduced at the working face, which consists of surface and underground equipment. Ten geophones are installed in the conveyor roadway and nine geophones are installed in the ventilation roadway of the underground working face. Signals collected by the geophones are received by two acquisition substations and transmitted to a surface computer terminal for data processing and analysis. The layout of the monitoring system is shown in Figure 3.

### 3.3. MS Monitoring Analysis of Coal Seam Floor

It should be noted that the detected MS events may originate not only from mining-induced disturbances but also from various factors such as stress redistribution, geological structural activities, and hydraulic disturbances. Regional stress adjustment is mainly manifested as the redistribution of deep rock stress under mining-induced conditions; structural activity is associated with local slippage within geological weak zones such as faults and folds; and hydraulic disturbances arise from pore-water pressure variations and seepage effects. Considering these factors comprehensively, the present study primarily utilizes spatial MS data from the floor to ensure that the selected data accurately reflect the fracture evolution and crack propagation characteristics of the floor rock mass.

**Figure 4:**
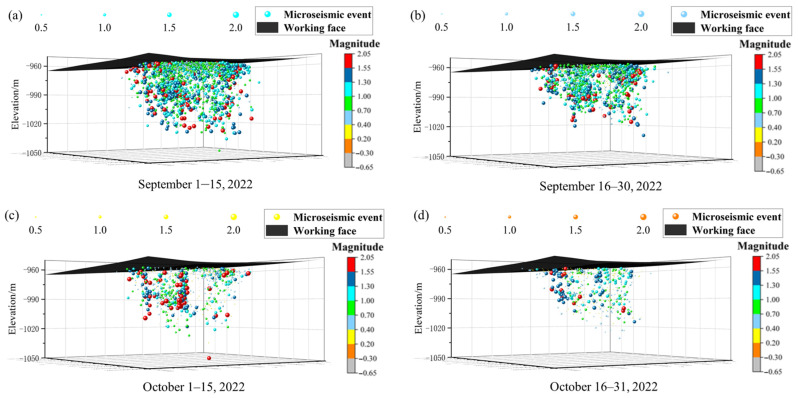
Spatial morphology of floor fracturing in the F.17-33200 working face. (**a**) Distribution of MS events from 1 to 15 September 2022; (**b**) Distribution of MS events from 16 to 30 September 2022; (**c**) Distribution of MS events from 1 to 15 October 2022; (**d**) Distribution of MS events from 16 to 31 October 2022.

To analyze floor rock mass fracturing and potential water inrush hazards in the F.17-33200 working face, a three-dimensional spatial distribution of MS events is plotted, through which the approximate location of floor failure depth is determined. Taking September and October 2022 as examples, the spatial distribution characteristics of floor MS events are shown in Figure 4. The size and color of the spheres represent the magnitude of MS events. The formula for calculating the Richter magnitude is presented as follows [35].

(1) $$M=\frac{\mathrm{lg}E}{b}+a$$

where *M* represents the Richter magnitude, *E* represents energy, *a* and *b* are constants, with *a* set to −0.15 and *b* set to 2.61 based on field experience.

As shown in Figure 4, the MS activity in the study area is dominated by events of moderate to low magnitude, although higher-magnitude events also occur. Statistical results indicate that a total of 5456 events with magnitudes below 1.30 (excluding the deep blue and red spheres) account for 89.27% of all recorded events. Mining activities at the working face cause stress concentration in the coal seam floor, and rock fractures occur when the rock strength is exceeded. The frequency of MS events is higher when closer to the coal seam floor, with the number of fractures decreasing vertically as depth increases. The overall cross-sectional profile exhibits an inverted trapezoid distribution, characterized by a wider upper part and a narrower lower part. The spatial distribution of MS events indicates that floor fracture locations are primarily concentrated within the 0–40 m depth range of the floor rock layer, with the deepest reaching 84.78 m below the floor. This indicates that significant floor rock activity occurs throughout the mining process in this area. As the depth of floor rock fractures in the coal seam increases, confined water in the limestone may potentially migrate upward along water-conducting channels, leading to floor water inrush.

## 4. Correlation Theoretical Model of MS-Water Inrush Volume

One of the necessary conditions for the occurrence of mine water inrush disasters is the formation of an inrush channel, that is, the damage-induced fracturing of the floor has already penetrated. As the working face advances, the coupling of mining-induced stress and confined water pressure causes floor fractures to exhibit typical nonlinear evolution and multi-scale expansion characteristics. Once the fractures gradually develop into a connected network, the aquifer water level begins to decline, followed by high-water-inflow events. According to the mechanism of floor water inrush, the predicted formula for aquifer water inrush volume is given as follows [36]:

(2) $$Q=\frac{\pi d^{2}}{4}L^{0.556\ln\frac{\rho^{0.2}d^{1.2}g}{0.092L\mu^{0.2}}}\left(H_{1}-H_{2}\right)$$

where *Q* represents the water inrush volume (m^3^/s), *d* is the diameter of the water inrush channel (m), *L* is the length of the water inrush channel (m), *ρ* denotes the density of water (kg/m^3^), *μ* is the dynamic viscosity of water (m^2^/s), *g* represents the gravitational acceleration (m/s^2^), *H*_1_ is the water head height before inrush (m), and *H*_2_ is the water head height after inrush (m).

During the formation of the above-mentioned water inrush channel, numerous fracture events continuously occur. As a novel physical monitoring technology, MS can effectively capture the initiation and evolution of floor fractures in coal seams. Signals generated by rock mass fracturing are received by more than four geophones, converted into data signals through underground equipment, and subsequently processed by surface instruments to perform source inversion, yielding the spatial coordinates (*x*, *y*, *z*). The MS system deployed at the F.17-33200 working face has been applied in multiple mines and is characterized by relatively high positioning accuracy. The spatial location of an MS event is taken as the center of a sphere, with the crack radius serving as the sphere’s radius. The diameter of the apparent volume of an MS event (the extent of the damage zone enveloped by the spherical failure region) and the spatial distance between two seismic sources are given by the following formulas [37].

(3) $$d_{1}=2\sqrt[{3}]{\frac{3M_{i}}{8\pi \sigma_{A}}}$$

(4) $$L_{1}=\sqrt{{\left(x_{2}-x_{1}\right)}^{2}+{\left(y_{2}-y_{1}\right)}^{2}+{\left(z_{2}-z_{1}\right)}^{2}}$$

where *M_i_* represents the seismic moment of the MS event, *σ_A_* represents the apparent stress, and *x*, *y*, *z* represent the spatial coordinates of the MS event.

**Figure 5:**
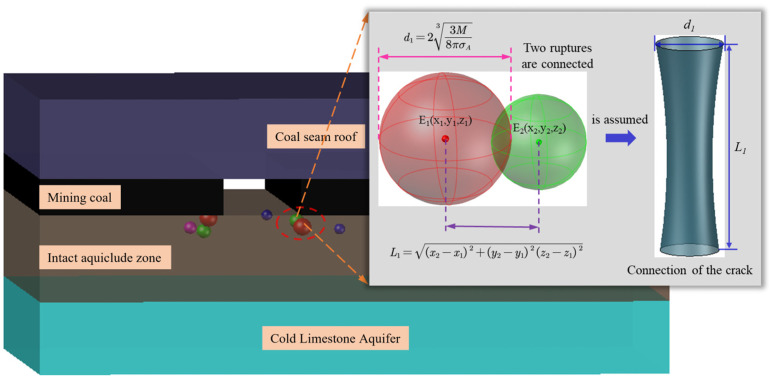
Model of rock crack connectivity (taking two seismic sources as an example).

Under the initial mining disturbance at the working face, stress concentration in the coal seam floor rock mass is relatively weak, and a small number of MS events begin to occur. Taking two MS events as an example, it is assumed that these two rock fracture events connect to form a crack. Consequently, the diameter of the fracture sphere can be approximated as the diameter between the crack’s endpoints, and the spatial distance between the two seismic sources can be regarded as the crack’s length, as shown in Figure 5.

**Figure 6:**
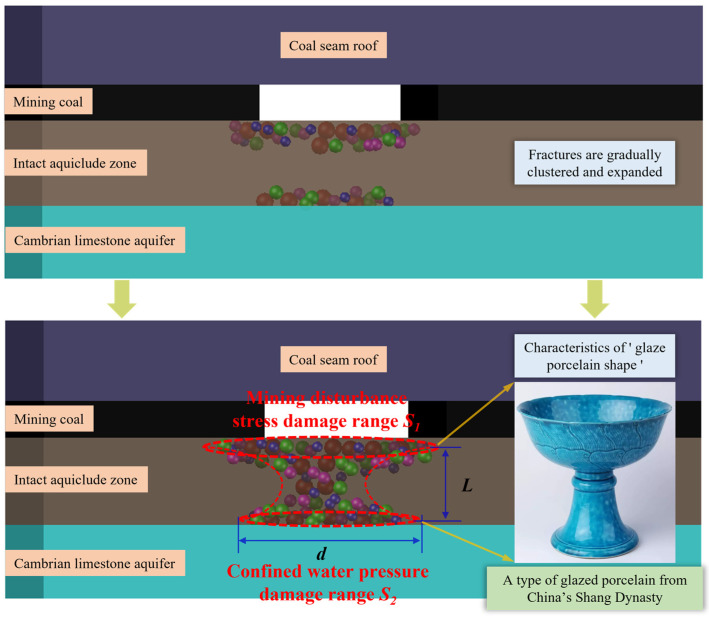
“Glazed porcelain shape” feature of the floor water inrush channel.

As the working face is continuously mined, the degree of floor failure progressively increases. The number and energy of MS events increase, their spatial extent expands, and a significant clustering pattern is observed. As shown in Figure 6, under the influence of mining-induced stress, MS events form a large damage zone (*S*_1_) in the upper part of the intact aquiclude. Because the disturbance becomes stronger closer to the working face, the extent of damage also enlarges, ultimately presenting a distribution connectivity characterized by a wide upper part and a narrow middle section. Meanwhile, under the confined water pressure, MS events also form a large damage zone (*S*_2_) in the lower part of the aquiclude. Because the water pressure increases closer to the aquifer, the extent of damage is greater, ultimately displaying a distribution connectivity characterized by a narrow middle section and a wide lower part.

The combination of *S*_1_ and *S*_2_ produces a morphology that is wide at both ends and narrow in the middle, resembling a high-footed glazed porcelain vessel from the Shang Dynasty of China. Under the combined action of mining-induced stress and confined water pressure, once the load-bearing capacity of the intact aquiclude is exceeded, the working face floor is penetrated by fracturing, resulting in the formation of a “glazed porcelain shape” water inrush channel. In contrast, if the load-bearing capacity of the aquiclude is not exceeded, only an “glazed porcelain shape” upper feature may form at the floor.

When the damage zones *S*_1_ and *S*_2_ in Figure 6 are each considered as a single entity, their connectivity mechanism is identical to that of the two MS events in Figure 5. Consequently, the diameter of the damage zone can be approximated as the diameter of the water inrush channel, and the spatial distance between the two damage zones *S*_1_ and *S*_2_ can be regarded as the length of the water inrush channel. By introducing correction coefficients *u* and *v*, the diameter and length are expressed as *d* = *ud*_1_ and *L* = *vL*_1_, respectively. Substituting these into and integrating Equations (2)–(4) yields a preliminary relationship between MS activity and the aquifer water inrush volume:


(5) $$Q=\pi u^{2}{\left(\frac{3M}{8\pi \sigma_{A}}\right)}^{\frac{2}{3}}{\left(v\sqrt{{\left(x_{2}-x_{1}\right)}^{2}+{\left(y_{2}-y_{1}\right)}^{2}+{\left(z_{2}-z_{1}\right)}^{2}}\right)}^{0.556\ln\frac{\rho^{0.2}d^{1.2}g}{0.092L\mu^{0.2}}}\left(H_{1}-H_{2}\right)$$

In summary, under constant hydrogeological conditions such as water density and viscosity, a certain relationship is observed between coal seam floor water inrush and MS activity. Moreover, the magnitude of aquifer water level fluctuations directly affects the volume of water inrush; therefore, a correlation also exists between MS activity and aquifer water levels.

## 5. Synergistic Effects of Precursory Indicators for Floor Water Inrush

### 5.1. Statistical Analysis of Floor MS Data

**Figure 7:**
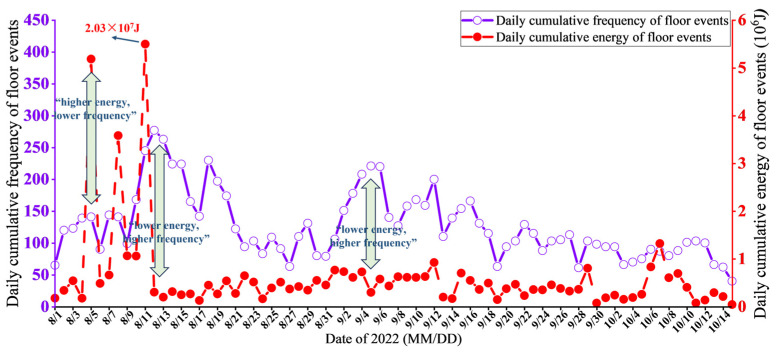
Analysis of MS energy and frequency in the floor of Pingdingshan No. 10 Coal Mine.

The daily cumulative energy and frequency of floor MS events from 1 August 2022, to 15 October 2022, are analyzed, revealing that during this period, both high-energy events with lower frequency and low-energy events with higher frequency are observed, as shown in Figure 7. This indicates that no definitive correlation exists between daily cumulative energy and frequency. However, compared to frequency, energy better characterizes the degree of rock fractures and the magnitude of risk; thus, this study focuses on analyzing the response characteristics between MS energy and aquifer water level.

In this study, “single high-energy events” are defined as individual MS events with energy exceeding 1 × 10^5^ J. This threshold is determined based on the statistical results of MS energy distribution at the Pingdingshan No. 10 Coal Mine and is used to characterize the strong instantaneous fracturing behavior of the surrounding rock under mining-induced stress conditions. “Higher daily cumulative energy” refers to the total energy accumulated from MS events within a single day that is significantly higher than the average level, reflecting an overall enhancement of rock fracturing activity on that day. For example, on 5, 8, and 11 August, the daily cumulative energies reached 5.19 × 10^6^ J, 3.58 × 10^6^ J, and 2.03 × 10^7^ J, respectively, indicating that the overall damage to the coal seam floor intensified during this period. The development and propagation of fractures were enhanced, and the risk of water inrush increased significantly.

### 5.2. Correlation Analysis of Single High-Energy Events and Water Level

**Figure 8:**
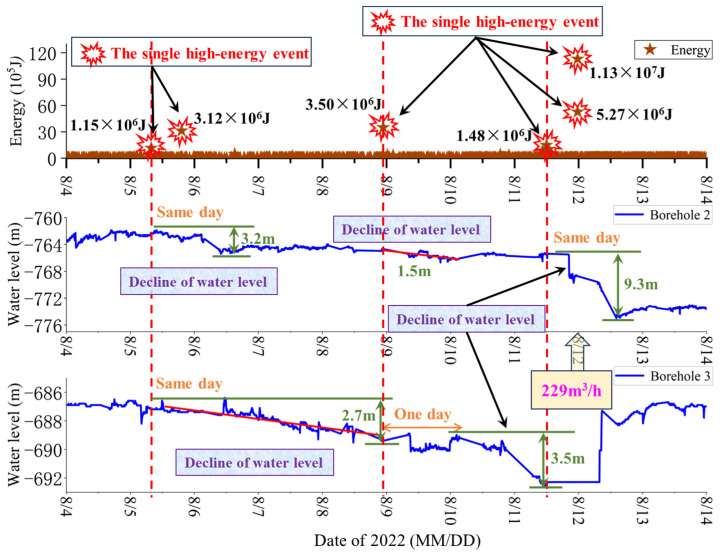
Correlation between single high-energy events and water level in Pingdingshan No. 10 Coal Mine.

Analysis of daily data reveals that single high-energy events in August result in elevated daily cumulative energy; thus, the correlation between single high-energy events and monitoring borehole water level changes at Pingdingshan No. 10 Coal Mine during this period is analyzed. Since monitoring Borehole 1 is used for water drainage protection during this period, the relationship between MS energy and water level changes in the other two monitoring boreholes is investigated based on field monitoring data from 4 August to 11 August, as shown in Figure 8.

As shown in Figure 8, two single high-energy events occur on 5 August, with energy magnitudes of 1.15 × 10^6^ J and 3.12 × 10^6^ J, respectively. The water level in Borehole 2, initially at −762.1 m, begins to decline slowly after the first high-energy event. After the second high-energy event on the same day, the water level decreases significantly to −765.3 m, resulting in a total decline of 3.2 m. The water level in Borehole 3, initially at −686.7 m, continues to decline slowly after the first high-energy event, reaching −689.4 m, with a total decline of 2.7 m. On August 8, a single high-energy MS event with an energy of 3.50 × 10^6^ J is observed in the floor of the working face. Subsequently, the water level in Borehole 2 begins to decline, dropping from −764.7 m to −766.2 m, with a total decline of 1.5 m. The water level in Borehole 3, still unstable due to the influence of the two high-energy events on August 5, begins to decline significantly after the third high-energy event. It drops from −689.3 m to −692.8 m, with a total decline of 3.5 m. Based on field monitoring data, a high-water-inflow event of 229 m^3^/h is recorded underground on 12 August.

### 5.3. Correlation Analysis of High Daily Cumulative Energy and Water Level

**Figure 9:**
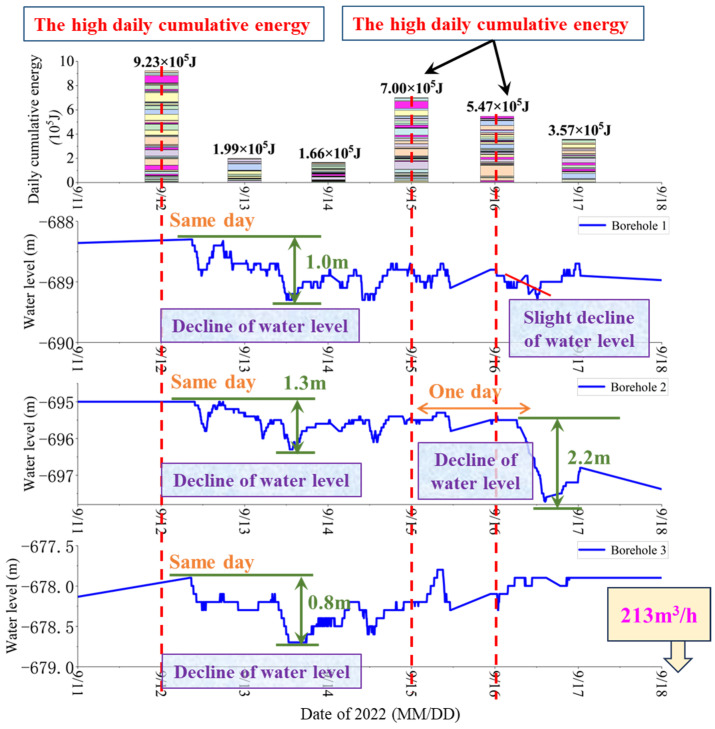
Correlation between high daily cumulative energy and water level in Pingdingshan No. 10 Coal Mine.

Analysis of September monitoring data reveals that floor MS events during this period exhibit relatively uniform energy magnitudes, with no single exceptionally high-energy event observed. Consequently, the relationship between high daily cumulative energy and water level changes is analyzed [38]. Based on monitoring data from 11 September to 18 September, graphs are plotted over time, as shown in Figure 9.

As shown in Figure 9, a high daily cumulative energy event occurs on 12 September, with an energy magnitude of 9.23 × 10^5^ J. Following this high daily cumulative energy event, the water level in Borehole 1 drops from −688.3 m to −689.3 m, with a total decline of 1.0 m; the water level in Borehole 2 drops from −695.0 m to −696.3 m, with a total decline of 1.3 m; and the water level in Borehole 3 drops from −677.9 m to −678.7 m, with a total decline of 0.8 m. Analysis reveals that, despite the different spatial distributions of Boreholes 1, 2, and 3, the timing and magnitude of their water level declines are consistent, with all exhibiting a slight decline on the same day. Furthermore, high daily cumulative energy events occur on 15 September and 16 September, with energy magnitudes of 7.00 × 10^5^ J and 5.47 × 10^5^ J, respectively. The water level in Borehole 2 responds with greater sensitivity, beginning to decline one day later, dropping from −695.5 m to −697.7 m, with a total decline of 2.2 m. Based on field monitoring data, a high water inflow event of 213 m^3^/h is recorded underground on 18 September.

In summary, the monitoring data from Pingdingshan No. 10 Coal Mine further reveal the sensitivity characteristics prior to floor water inrush: significant correlations are identified between single high-energy events and high daily cumulative energy of MS activity, and water level decline. Therefore, a combined MS energy-water level approach is applied for the early warning of floor water inrush in Pingdingshan No. 10 Coal Mine. When water level decline is accompanied by high-energy MS characteristics, protective measures and water drainage exploration are required to be strengthened at the working face.

## 6. Construction and Field Verification of the Integrated Early Warning Model

### 6.1. Indicator Optimization Based on GA

**Figure 10:**
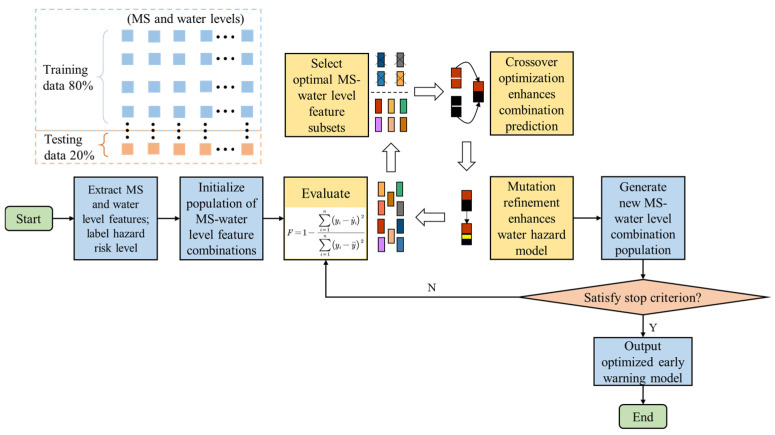
Flowchart of the GA training process.

Based on the aforementioned precursor correlation characteristics of floor water inrush, this section focuses on the construction of an integrated early warning system for floor water hazards. Using daily accumulation as the time window, the core MS indicators are selected as energy and frequency, while the water level indicators correspond to the level decline measured at three underground monitoring boreholes (Borehole 1, Borehole 2, and Borehole 3). Due to multiple factors, including the complex geological structure and mining disturbances, the sensitivity of each basic indicator to water inrush precursors varies. Therefore, advanced machine learning and optimization methods are introduced in this study. The response characteristics of MS and each monitoring borehole are systematically quantified using a GA, and the optimal indicator combination for early warning is ultimately selected, as shown in Figure 10.

(1) A multi-source indicator dataset {*x*_1_, *x*_2_, …, *x_n_*, risk level} for floor water hazards is first constructed, and the dataset is separated into MS-water level features *x*_1_–*x*_n_ and the risk label, risk level. The risk level is determined based on on-site hazard investigations and comprehensive water inflow assessment. The indicator population parameters are then standardized using ‘StandardScaler’, and the monitoring data are divided into 80% training set and 20% testing set.

(2) In terms of feature selection, the GA iteratively optimizes the MS-water level feature combinations by simulating the processes of natural selection and genetic variation, so that the most predictive subset is selected from the candidate indicators. The model’s goodness of fit, *F*, is typically evaluated using the following fitness function [39]:

(6) $$F=1-\frac{\sum_{i=1}^{n}{\left(y_{i}-{\hat{y}}_{i}\right)}^{2}}{\sum_{i=1}^{n}{\left(y_{i}-\bar{y}\right)}^{2}}$$

where *y_i_* denotes the true label of the *i*-th sample, $\hat{y_{i}}\;$represents the predicted floor water risk, $\bar{y}$ is the mean of the true labels, and *n* is the number of samples from Pingdingshan No. 10 Coal Mine.

In this study, a GA framework is constructed based on the DEAP library. By iteratively optimizing feature combinations, the feature space dimensionality is effectively reduced while the model prediction accuracy is improved. The algorithm hyperparameters are set as follows: population size of 50, number of iterations of 50, crossover probability of 0.7, mutation rate of 0.05, and feature number of 5. The top ten indicator combinations from the training results are presented in Table 1. The results indicate that the MS energy-water level combination outperforms the frequency-water level combination, indirectly confirming the relative advantage of MS energy in floor water hazard applications. Among them, the combination of MS energy, Borehole 1, Borehole 2, and Borehole 3 achieves a high goodness-of-fit, F, of 0.6860 in the floor water inrush application at Pingdingshan No. 10 Coal Mine, indicating that this feature combination possesses strong predictive reliability.

### 6.2. Importance Weight Allocation Based on AHP and RF

#### 6.2.1. Construction of the Hierarchical Model

**Figure 11:**
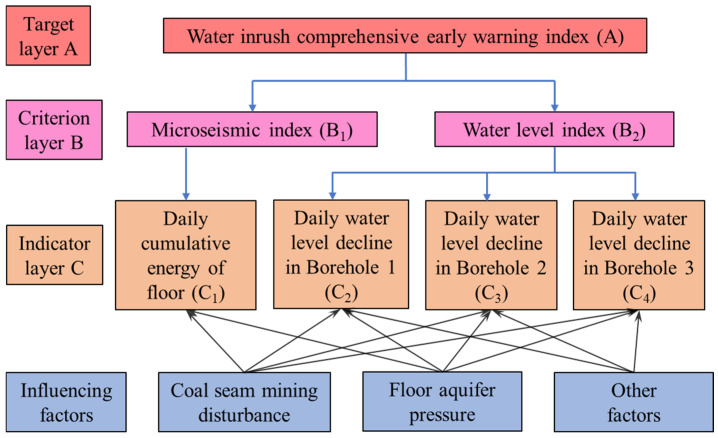
Hierarchical analytic structure model.

In this study, the structured judgment advantages of AHP are innovatively integrated with the nonlinear modeling capability of RF to achieve a scientific and rational allocation of weights for water hazard prediction indicators. First, a hierarchical model is constructed based on the optimal results of the GA, as shown in Figure 11. The water inrush comprehensive early warning index A is defined as the target layer of the model; the criterion layer consists of the MS index B_1_ and the water level index B_2_; the indicator layer includes daily cumulative energy of floor C_1_, daily water level decline in Borehole 1 C_2_, daily water level decline in Borehole 2 C_3_, and daily water level decline in Borehole 3 C_4_; the influencing factors include coal seam mining disturbance, floor aquifer pressure, and other factors.

Within the same level, the relative importance among elements is determined through expert judgment or questionnaire surveys [40], and a pairwise comparison matrix is then constructed accordingly. Each element of the matrix represents the relative importance or comparative preference between two criteria or alternatives. To ensure the scientific validity and rationality of expert judgments, a consistency test is required. First, the consistency index (CI) is calculated, and the random consistency index (RI) is introduced for comparison. When the consistency ratio (CR) is less than 0.10, the judgment matrix is considered acceptable, and its normalized eigenvector can be adopted as the weight vector; otherwise, a re-evaluation is required.

#### 6.2.2. RF Model Construction

**Figure 12:**
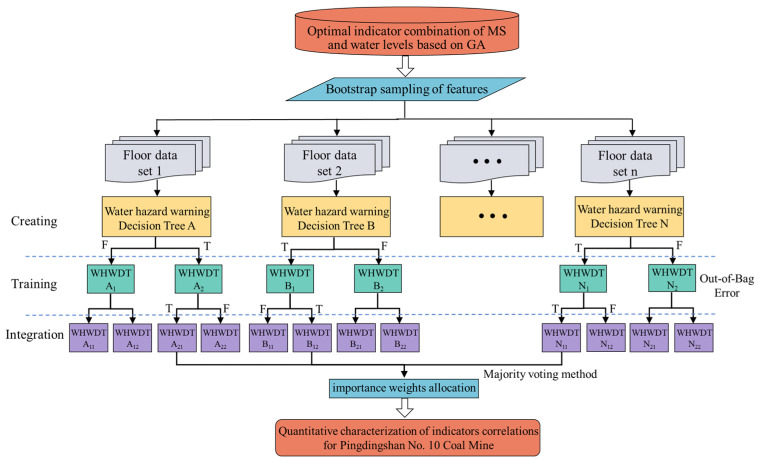
RF model architecture.

Similarly, based on the optimal MS-water level indicator combination from GA, RF constructs and integrates multiple decision trees, with the average of each tree’s prediction results as the final output. This method has strong capabilities in handling high-dimensional data and nonlinear relationships, and the model architecture is shown in Figure 12.

The RF model is trained to extract the “importance scores” of each feature, reflecting the contribution of each feature to the model’s floor water hazard prediction results, and these scores are used to assign weights in the optimal indicator combination. The importance scores are calculated by evaluating the contribution of each feature to reducing the mean square error (MSE) at the split nodes, as shown in Equation (7) [41]:

(7) $$I(X_{i})=\frac{1}{T}\sum_{t=1}^{T}\sum_{n\in nodes(t)}\left(\Delta MSE(X_{i},n)\cdot w_{t,n}\right)$$

where *T* represents the number of trees in the forest, set to 100, *nodes*(*t*) refers to all split nodes in the *t*-th tree, Δ*MSE*(*X_i_*,*n*) represents the reduction in MSE due to feature *X_i_* at node *n*, and *w_t,n_* is the weight of node *n*.

**Figure 13:**
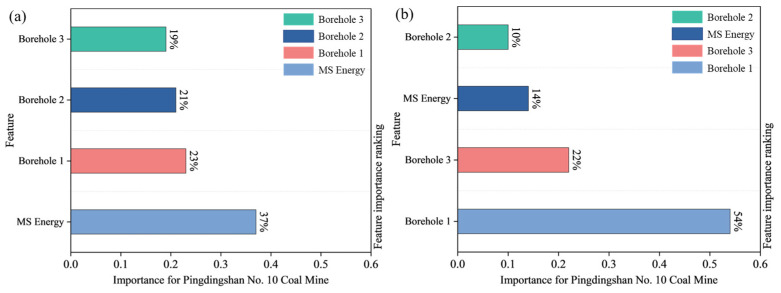
Indicator weight allocation results. (**a**) AHP importance assessment; (**b**) RF importance assessment.

This weight allocation method based on AHP and RF combines the advantages of both subjective and objective approaches. The former incorporates expert knowledge to ensure logical consistency, while the latter relies on data-driven approaches to reveal the importance of nonlinear features. This combined method significantly enhances the interpretability of the indicator combination and helps identify the key factors contributing to water hazard risk. The feature evaluation results are shown in Figure 13.

The consistency test result in AHP is CR = CI/RI = 0.024 < 0.10, indicating that the judgment matrix has good consistency. At the same time, the water hazard indicator combination performs excellently in the RF model, with mean absolute error (MAE) and root mean square error (RMSE) values of 0.027 and 0.046, respectively. Given that the product method has advantages in consistency amplification and non-compensatory aspects, this study employs this method to integrate the weight results of AHP and RF, highlighting the important features jointly determined by both methods and suppressing the bias introduced by each individual method, as shown in Equations (8) and (9). The weights for MS energy and water level Boreholes 1, 2, and 3 in Pingdingshan No. 10 Coal Mine are calculated to be 22%, 52%, 9%, and 17%, respectively, thereby achieving a quantitative representation of the correlation of floor water inrush precursor indicators.

(8) $${\tilde{w}}_{i}=w_{i}^{\mathrm{AHP}}\cdot w_{i}^{\mathrm{RF}}$$

(9) $$w_{i}^{*}=\frac{{\tilde{w}}_{i}}{\sum_{i=1}^{4}{\tilde{w}}_{i}}$$

where $w_{i}^{AHP}$ and $w_{i}^{RF}$ represent the AHP and RF weights for the *i*-th floor water hazard feature, respectively, and $w_{i}^{*}$ is the final normalized weight for the MS-water level.

### 6.3. Field Engineering Validation

#### 6.3.1. Classification of Water Inrush Risk Levels

The optimal indicator combination for floor water inrush prediction based on GA, along with the weights of each indicator determined through AHP and RF, have been obtained. On this basis, the MS-water level monitoring data is standardized, and a linear weighting method is used to calculate the daily comprehensive water inrush risk value. The higher the comprehensive early warning index of the working face, the greater the water inrush risk on that day. The structural stability and water inflow measured in previous field tests are used as evaluation standards, categorizing the water inrush risk of the working face into weak risk level I (0 ≤ R ≤ 0.3), moderate risk level II (0.3 < R ≤ 0.6), and high risk level III (0.6 < R ≤ 1). Risk level I corresponds to the overall stability of the floor aquiclude, with no impact on mining operations, allowing the working face to proceed as planned. Risk level II indicates potential water inrush hazards, with uncertainty in the risk, requiring enhanced water drainage and protection measures. Risk level III indicates a significant water inrush risk, triggering an alarm at the working face, halting operations, and initiating emergency measures.

#### 6.3.2. Engineering Application Effect Analysis

**Figure 14:**
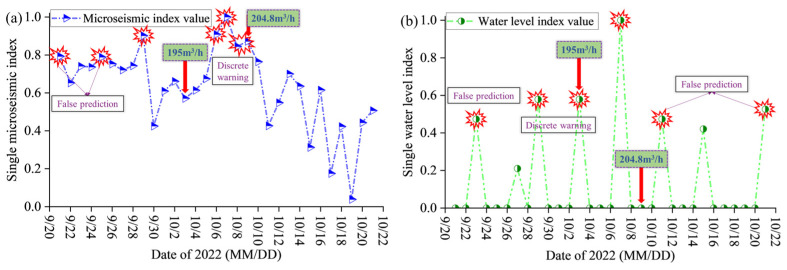
Application analysis of univariate index at Pingdingshan No. 10 Coal Mine. (**a**) MS index early warning effect; (**b**) Water level index early warning effect.

**Figure 15:**
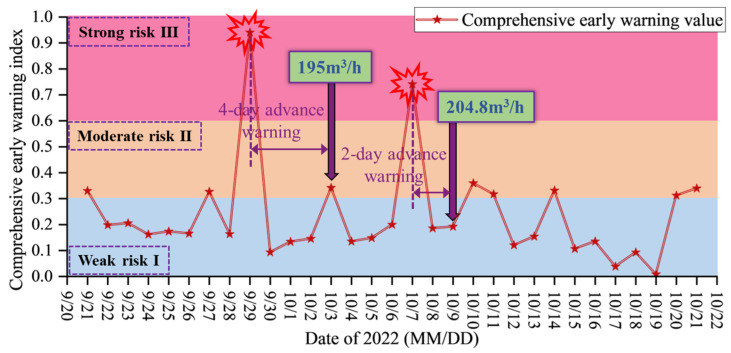
Application effect analysis of comprehensive index at Pingdingshan No. 10 Coal Mine.

To verify the effectiveness of the basic and comprehensive indices in floor water inrush early warning in coal seams, a subsequent continuous period during the production of the 17-33200 working face at Pingdingshan No. 10 Coal Mine is selected for validation. The collected data includes MS monitoring results, water level changes in monitoring boreholes, and actual water inrush volume. The calculation results of the MS-water level univariate index and the comprehensive index are shown in Figure 14 and Figure 15.

The univariate index exhibits a certain predictive capability for water inrush at the working face, but its early warning performance has two main shortcomings: ① High values of the daily cumulative index do not necessarily correspond to high-water-inrush events at the working face. As shown in Figure 14a,b, although the MS index exhibited high values on 21 September and 25 September, and the water level index showed high values on 23 September, 11 October, and 21 October, no significant water inrush events occurred afterward. The single-dimensional MS index and water level index, respectively, produced false-positive warnings on two and three days. ② Different univariate indices may provide inconsistent predictions, thereby reducing the overall accuracy of early warning. As shown in Figure 14a, prior to the high-inrush event of 204.8 m^3^/h on 9 October, the MS index presented discrete warning results between 6 and 9 October. Similarly, as shown in Figure 14b, before the high-inrush event of 195 m^3^/h on 3 October, the water level index also produced discrete warning results on 29 September and 3 October. From the above analysis, it can be concluded that the univariate index only reflects partial precursor information and is prone to false alarms in prediction and early warning.

Based on the water inrush risk classification results, the comprehensive index values for this period are partitioned. When the value exceeds *R*_0_ = 0.60, corresponding to the high-risk level III region, an alarm is triggered at the working face. As shown in Figure 15, the comprehensive index values are calculated to have exceeded the alarm threshold *R*_0_ on 29 September and 7 October, with magnitudes of 0.94 and 0.74, respectively. The water inrush early warning results indicate that the MS-water level comprehensive index successfully provides warnings for high-water-inrush events within four days. In cases where a single index shows high values but no significant water inrush events occur afterward, the comprehensive index does not exhibit high values on the corresponding dates. Engineering practice demonstrates that this method effectively integrates multiple types of sensitive precursor information, eliminates index discrepancies through heterogeneous fusion, and achieves accurate prediction and early warning of water inrush from coal seam floors.

On-site validation of the model is a crucial step for its promotion and application across different mining areas. Based on the measured results from Pingdingshan No. 10 Coal Mine, the reliability and adaptability of the model under actual mining conditions have been verified. All characteristic indicators of the model are derived from real-time monitoring data. The algorithm achieves adaptive weight distribution through the GA-AHP-RF fusion method, enabling the model to be applicable to mines with various similar geological and hydrological conditions. On-site results indicate that the model’s comprehensive indicators exceed the early-warning threshold within the 4 days prior to high water inrush, corresponding to the critical stage of floor fracture propagation and the formation of water inrush channels. Accurate early warnings are issued shortly before water inrush events, providing sufficient time for drainage, reinforcement, and risk mitigation in the mine. This significantly improves the precision and timeliness of water hazard management decisions, holding important engineering significance.

## 7. Conclusions

(1) Among the MS events recorded in the floor of the F.17-33200 working face, a total of 5456 events had magnitudes less than 1.30, accounting for 89.27% of all events. The frequency of MS events decreases vertically with increasing depth, with fracture events mainly concentrated within 0–40 m of the floor, and the deepest occurrence reaching 84.78 m.
(2) Two events are used as examples to characterize the intrinsic relationship between MS activity and fractures. By integrating the “glazed porcelain shape” feature of floor rock failure that forms water inrush channels, a theoretical expression of aquifer water inrush volume based on MS activity is derived. It is also shown that a certain correlation exists between MS activity and water level variations.
(3) Analysis of field monitoring data shows that significant sensitivity response characteristics exist prior to floor water inrush: single high-energy events and high daily cumulative energy of MS activity are found to be strongly correlated with water level decline. Based on this, a method that combines MS energy with water level dynamics is proposed, by which effective early warning of floor water inrush disasters can be achieved.
(4) The GA-AHP-RF model quantifies the relationships among sensitive indices, with CR, MAE, and RMSE values of 0.024, 0.027, and 0.046, respectively. The weights for floor MS energy and water levels in Boreholes 1, 2, and 3 are 22%, 52%, 9%, and 17%. Field results confirm that the comprehensive index can provide an accurate early warning up to four days before high water inrush events.

Although the effectiveness of the comprehensive early-warning system has been verified in this study, it remains constrained by the temporal-spatial scale of the data and geological variability. Moreover, improvements are still needed in the integration of temporal-spatial-magnitude information of floor MS events, spatial visualization of fracture zones, and deep-learning-based prediction of water hazard risks. Future research should combine multi-working-face field monitoring with intelligent optimization to continuously enhance the model’s accuracy and adaptability under long-term dynamic conditions, thereby promoting the broader application of the early-warning system in complex geological environments.

## Figures and Tables

**Table 1:** Optimal combinations of indicators for water inrush prediction.

Indicator Combination	F-Value	Indicator Combination	F-Value
MS energy, Borehole 1, Borehole 2, Borehole 3	0.6860	MS energy, MS frequency, Borehole 3	0.5109
MS energy, Borehole 1, Borehole 3	0.6763	MS energy, Borehole 3	0.4636
MS energy, MS frequency, Borehole 1, Borehole 3	0.6757	Borehole 1, Borehole 3	0.4551
MS energy, MS frequency, Borehole 1, Borehole 2, Borehole 3	0.6399	MS frequency, Borehole 1, Borehole 2, Borehole 3	0.4541
MS energy, Borehole 1, Borehole 2	0.5134	MS frequency, Borehole 1, Borehole 3	0.4322

## Data Availability

Data will be made available on request.
